# Dataset of future-shifted weather files for Canada using climate projections from CMIP6

**DOI:** 10.1016/j.dib.2025.111667

**Published:** 2025-05-15

**Authors:** Stephen R. Sobie, Charles L. Curry

**Affiliations:** a Pacific Climate Impacts Consortium, University House 1, PO Box 1700 Stn CSC University of Victoria, Victoria, British Columbia, V8W 2Y2, Canada; b School of Earth and Ocean Sciences, Bob Wright Centre, PO Box 1700 STN CSC University of Victoria, Victoria, British Columbia, V8W 2Y2, Canada

**Keywords:** Climate change, Building energy modelling, Typical meteorological years, Canadian weather files

## Abstract

Investigating energy use in new building designs or existing structures in Canada is often performed with energy models that incorporate present-day climate information from the Canadian Weather Year for Energy Calculation 2020 (CWEC2020) weather files. Here we present a new dataset of future-shifted versions of these weather files that have been produced at all CWEC2020 sites across Canada, incorporating projections from the latest generation of climate models from CMIP6. These future-shifted files have been generated using a weather file “morphing” procedure applied to adjust hourly time series of selected thermodynamic variables including dry bulb and dew point temperature, relative humidity, and surface pressure. Projected changes used to calculate morphing factors were taken from CMIP6 global climate models following low, medium and high future emissions pathways (SSP1 2.6, SSP2 4.5, SSP5 8.5). Using the projections from each pathway, future-shifted files have been produced for five future periods from the 2040s through the 2080s. These files facilitate the use of energy modelling to understand building performance and guide design choices for infrastructure under future climate change. All of the future-shifted CWEC2020 files are publicly available via the Pacific Climate Impacts Consortium (PCIC) Weather Files Data Portal at https://www.pacificclimate.org/data/weather-files

Specifications Table

**Table:** 

Subject	Earth & Environmental Sciences
Specific subject area	Meteorological weather files incorporating future projections of climate change for use in building energy modelling.
Type of data	Hourly weather files in both EnergyPlus Weather (EPW) and comma separated value (csv) format for five future time periods under low, medium, and high emissions scenarios.
Data collection	**CWEC2020 Files:** Canadian Weather Year for Energy Calculation (CWEC) weather files at 564 sites were acquired from the Engineering Climate Services Unit at Environment and Climate Change Canada [1]. **CMIP6 Models:** Future projections from 18 models in the Sixth Coupled Model Inter-comparison Project (CMIP6; [2]) following Shared Socioeconomic Pathways (SSPs) for low (SSP1 2.6), moderate (SSP2 4.5) and high (SSP5 8.5) future emissions were obtained from the Earth System Grid Federation data portal. **CMIP6 Downscaled Temperatures:** Statistically downscaled future projections of daily maximum and minimum temperatures for Canada were obtained from the Canadian Downscaled Climate Scenarios - Univariate CMIP6 (CanDCS-U6) data portal [3].
Data source location	CWEC2020 Weather Files: Environment and Climate Change Canada [1] https://open.canada.ca/data/en/dataset/55438acb-aa67–407a-9fdb-1cb21eb24e28 CMIP6 Models: Earth System Grid Federation data portal [2] https://esgf.github.io/nodes.html CMIP6 Downscaled Temperatures: PCIC CanDCS-U6 data portal [3] https://www.pacificclimate.org/data/statistically-downscaled-climate-scenarios
Data accessibility	Data repository name: Zenodo Direct URL to the repository: https://doi.org/10.5281/zenodo.15151116
Related research article	None

## 1 Value of the Data

• These future-shifted weather files enable analysis of building performance under climate conditions that are expected to change over the lifespans of many structures.
• The dataset is provided at all existing CWEC2020 locations in Canada using the standard EnergyPlus Weather file (EPW) format employed by energy modelling software, as well as in comma separated value (csv) format for other applications. The future-shifted files can be implemented into current methods and used to compare building performance in future climates.
• The files are provided for multiple time intervals using three future emissions scenarios, enabling the assessment of potential ranges of future building performance under different magnitudes of projected climate change.

## 2 Background

Current weather files in Canada (CWEC2020 files; [1]) offer a summary of typical climatic conditions at specific locations to analyze and simulate building energy use. These files contain hourly weather station-based observations encompassing a single year, defined as a Typical Meteorological Year (TMY; [4]), that is meant to be representative of average conditions from the recent past (1998–2017). Ongoing climate change means the information summarized in these weather files may not reflect present day conditions and is expected to become increasingly unrepresentative over typical building lifespans as warming and other changes continue [5]. Various methodologies have been applied to incorporate climate change projections into weather files [6], with a “morphing” procedure that adjusts the mean and variance of the hourly series being a common approach [7]. Future-shifted files generated with this method can be produced using projected changes calculated from climate models for a range of future periods. In combination with present day CWEC2020 files, the future-shifted files can then be used with energy modelling software to compare building energy use between past and future conditions.

## 3 Data Description

**Fig. 1:**
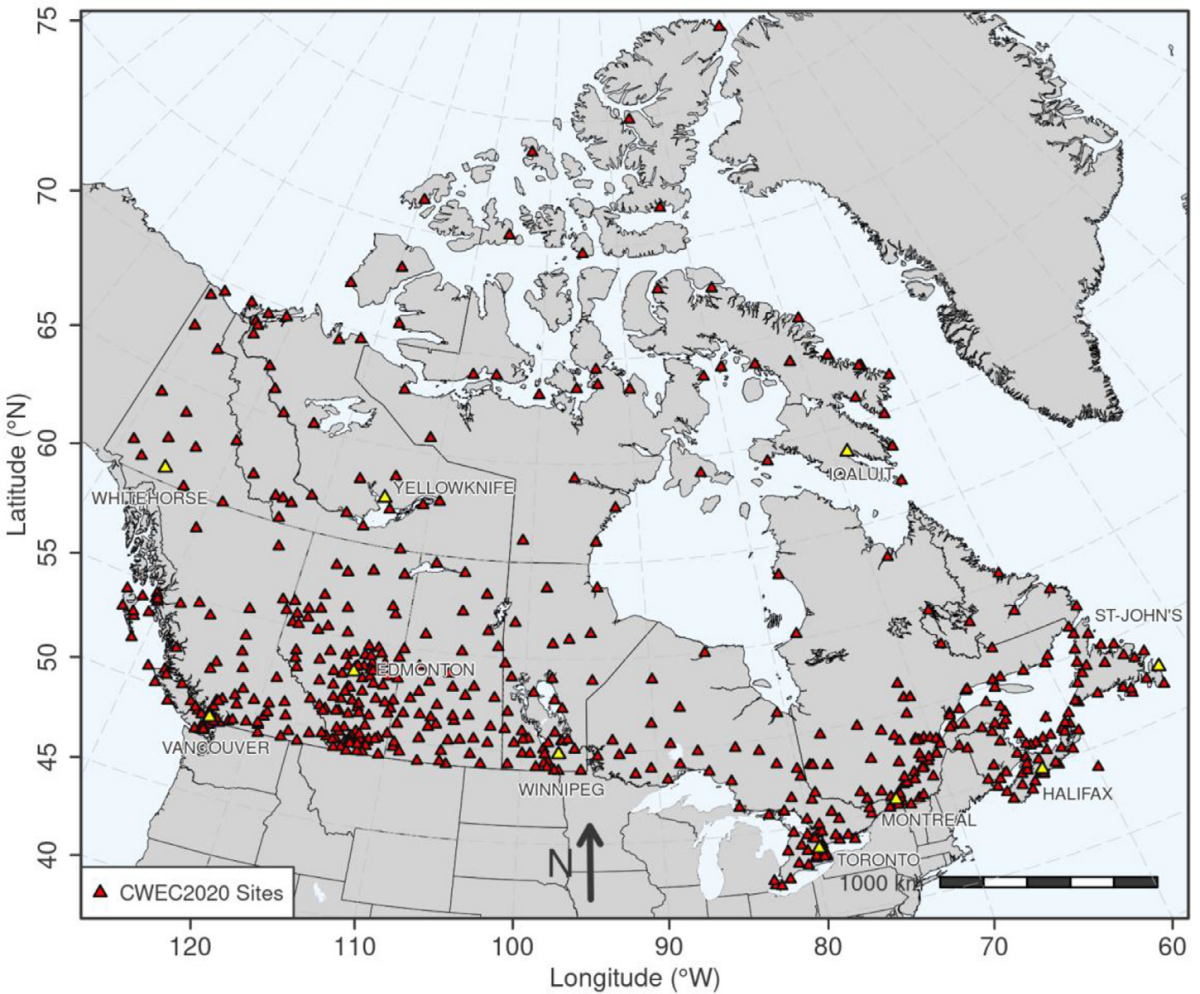
Map of the 564 CWEC2020 locations in Canada, identified by red triangles. The locations of major cities are indicated by yellow triangles.

The dataset consists of 15 future-shifted weather files at each of the 564 CWEC2020 locations in Canada, which are shown in Fig. 1. Future-shifted files employ the same EPW file structure formatting as the current CWEC2020 files. Within each future-shifted file, the hourly time series of dry bulb and dew point temperature, relative humidity, and surface pressure are adjusted using the morphing method to incorporate future climate change. The hourly time series for all other variables remain unchanged. In addition to the hourly time series adjustments, the “Comment Lines” 1 and 2 in the header block of each EPW file have been modified to include information about the morphing applied to create the future-shifted files. The text added to Comment Line 1 includes both the time period and emissions scenario of the projections used to create the future-shifted series. The line also includes the web link to the data portal at which all files can be accessed. The text added to Comment Line 2 lists the variables modified, the file version number, and the file creation date. All other header information in the files is unchanged. All future-shifted CWEC2020 EPW files are accessible via a purpose-built data portal (https://www.pacificclimate.org/data/weather-files) with search options to select files from specific locations. Files can be filtered according to province, coordinates, elevation, time interval, or emissions scenario. Once a future-shifted file is selected, it can be downloaded directly using the web link provided by the portal.

## 4 Experimental Design, Materials and Methods

Future versions of existing CWEC2020 weather files are produced by modifying the time series of hourly values within the files using projections from both CMIP6 global climate models (GCMs) and statistically downscaled CMIP6 scenarios of temperature. Existing hourly series in the weather files are modified using additive or multiplicative morphing factors that are derived from the climate projections. For each day of the year, these factors are calculated for a future climatological period (e.g. 2050s) relative to a 1991–2020 historical period (encompassing the various CWEC2020 baseline periods between 1998–2017). Morphing factors are smoothed with a 21-day moving average before being applied to the CWEC2020 hourly series to produce future-shifted versions. This approach is adapted from Belcher et al., 2005 [7] using morphing factors applied daily instead of monthly [8] to dry bulb temperature, dew point temperature, relative humidity, and surface pressure.

Morphing factors for dry bulb temperature are obtained from CMIP6 simulations that have been statistically downscaled using the Bias Correction/Constructed Analogues with Quantile delta mapping reordering method (BCCAQv2; [9]). These downscaled scenarios comprise part of the Canadian Downscaled Climate Scenarios-Univariate (CMIP6) dataset (CanDCS-U6; [3]). Using both downscaled daily maximum and minimum temperatures provides higher spatial detail to the temperature morphing factors, incorporating information from the Natural Resources Canada gridded meteorological dataset (NRCANmet; [10]) that is used in the downscaling procedure. When calculating dry bulb temperature morphing factors, projections from the CanDCS-U6 grid cells closest to each CWEC2020 location are used. To ensure that the same CMIP6 model ensemble is used to calculate all morphing factors, the downscaled temperature projections are obtained from the same GCM ensemble selected for the other GCM-based factors.

The CMIP6 GCMs used in producing future CWEC2020 files are selected from the ensemble of 26 GCMs used to produce the CanDCS-U6 dataset. Models from this ensemble for which the additional variables of specific humidity and air pressure are available from the Earth System Grid Federation data portal (ESGF; [11]) are selected to calculate morphing factors. The subset of GCMs satisfying these criteria includes 18 models from the CanDCS-U6 ensemble, which are listed in Table 1. In cases where more than one simulation is available for a GCM and emissions pathway, the first available realization (simulation) is selected. Dew point temperature and relative humidity variables (necessary to produce the morphing factors) are derived from the temperature, surface air pressure, and specific humidity variables [12]. Once the morphing factors are calculated for each GCM, the factors are bilinearly interpolated to 1/12° spatial resolution to match the resolution of the CanDCCS-U6 downscaled simulations. At each CWEC2020 site, morphing factors for the additional variables are then extracted from the nearest grid cell within each of the interpolated GCMs.

**Table 1:** List of 18 CMIP6 global climate models used to produce morphing factors for the CWEC2020 future-shifted weather files. The realization name denotes the specific simulation used from the GCM and represents the run “r”, initialization “i”, physics version “p”, and forcing “f”.

Institution	Model Name	Realization
CSIRO-ARCCSS (Australia)	ACCESS-CM2	r1i1p1f1
CSIRO (Australia)	ACCESS-ESM1–5	r1i1p1f1
CCCma (Canada)	CanESM5	r1i1p2f1
Euro-Mediterranean Centre for Climate Change (Italy)	CMCC-ESM2	r1i1p1f1
CNRM-CERFACS (France)	CNRM-CM6-1	r1i1p1f2
CNRM-CERFACS (France)	CNRM-ESM2–1	r1i1p1f2
EC-Earth-Consortium (Europe)	EC-Earth3	r4i1p1f1
EC-Earth-Consortium (Europe)	EC-Earth3-Veg	r1i1p1f1
Institute of Atmospheric Physics (China)	FGOALS-g3	r1i1p1f1
NOAA-Geophysical Fluid Dynamics Laboratory (USA)	GFDL-ESM4	r1i1p1f1
Met Office Hadley Centre (UK)	HadGEM3-GC31-LL	r1i1p1f3
Institute for Numerical Mathematics (Russia)	INM-CM4–8	r1i1p1f1
Institute for Numerical Mathematics (Russia)	INM-CM5–0	r1i1p1f1
Max Planck Institute for Meteorology (Germany)	MPI-ESM1–2-HR	r1i1p1f1
Max Planck Institute for Meteorology (Germany)	MPI-ESM1–2-LR	r1i1p1f1
Meteorological Research Institute (Japan)	MRI-ESM2–0	r1i1p1f1
Research Center for Environmental Changes (Taiwan)	TaiESM1	r1i1p1f1
Met Office Hadley Centre (UK)	UKESM1–0-LL	r1i1p1f2

Eq. (1) illustrates the additive scaling applied to observed hourly surface air pressure (P_h_*_,o_*) from the weather file. On the i*^th^* day of the year, the morphing factor obtained from the smoothed GCM projections for the same day *∆[P_d_(i)]* is applied to the 24 hourly values for that day P_h_*_,o_(i)* to produce hourly air pressure values for the future day P_h_*_,f_(i)*. An equivalent procedure is applied to variables modified using multiplicative factors such as relative humidity in which the daily relative scaling factor *α[H_d_]* is applied to the existing hourly values *H_h,o_* to produce future hourly values (*H_h,f_*; Eq. (2)).

(1) $$P_{h,f}\left(i\right)=P_{h,o}\left(i\right)+\Delta \left[P_{d}\left(i\right)\right]$$

(2) $$H_{h,f}\left(i\right)=H_{h,o}\left(i\right)\times \alpha \left[H_{d}\left(i\right)\right]$$

The morphing procedure for dry bulb temperature and dew point temperature uses both additive and multiplicative morphing factors. The additive factor is the projected change in average temperature for the i*^th^* day of the year *∆[T_d_(i)]* obtained from the CanDCS-U6 simulations. The relative factor *α[T_d_]* is calculated using projected changes in daily maximum temperature *∆[TX_d_(i)]* and minimum temperature *∆[TN_d_(i)]*, scaled by the diurnal range of observed hourly temperatures *T_h,o_*.

(3) $$\alpha \left[T_{d}\left(i\right)\right]=\frac{\Delta \left[TX_{d}\left(i\right)\right]-\Delta \left[TN_{d}\left(i\right)\right]}{\max\left[T_{h,o}\left(i\right)\right]-\min\left[T_{h,o}\left(i\right)\right]}$$

Future hourly temperature values *T_h,f_(i)* for the i*^th^* day are produced by combining these two factors to adjust the daily mean and anomalies of the existing temperature hourly series *T_h,o_(i)*.

(4) $$\begin{array}{c} T_{h,f}\left(i\right)=T_{h,o}\left(i\right)+\Delta \left[T_{d}\left(i\right)\right]+\alpha \left[T_{d}\left(i\right)\right]\left(T_{h,o}\left(i\right)-\bar{T_{h,o}\left(i\right)}\right) \end{array}$$

Dew point temperature values *D_h,o_(i)* are adjusted similarly, except that the relative morphing factor *α[D_d_(i)]* is calculated using the differences in past and future standard deviation of dew point temperature for each day of the year as the daily maximum and minimum dew point temperature values are not available from the climate model simulations used in this analysis.

(5) $$\alpha \left[D_{d}\left(i\right)\right]=\frac{\sigma D_{f}\left(i\right)}{\sigma D_{o}\left(i\right)}$$

(6) $$D_{h,f}\left(i\right)=D_{h,o}\left(i\right)+\Delta \left[D_{d}\left(i\right)\right]+\alpha \left[D_{d}\left(i\right)\right]\left(D_{h,o}\left(i\right)-\bar{D_{h,o}\left(i\right)}\right)$$

**Fig. 2:**
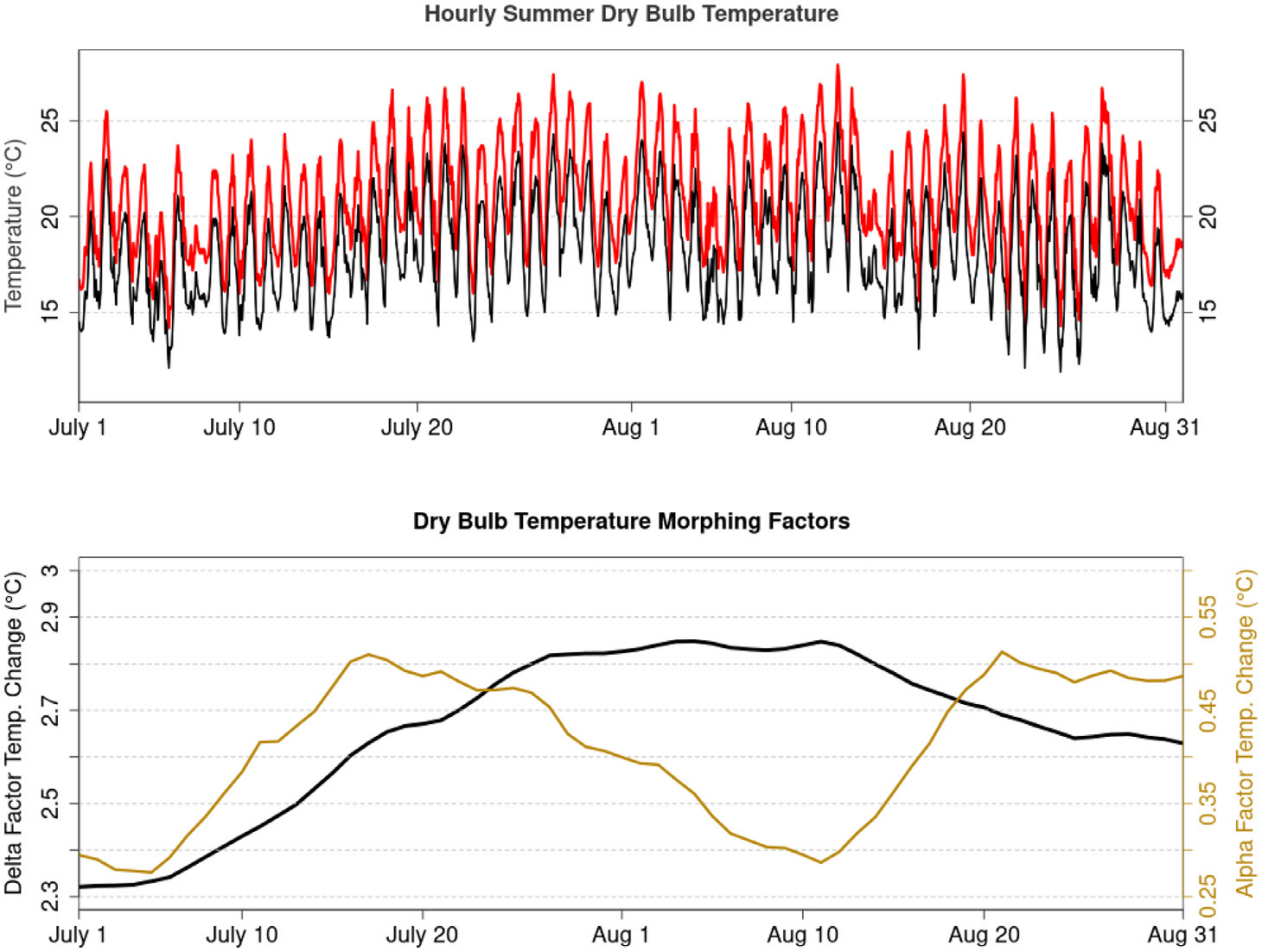
Illustration of the morphing method applied to hourly dry bulb temperature during July and August at Vancouver International Airport. The top panel displays hourly dry bulb temperatures between July 1st and August 31st from the CWEC2020 file (black line). The red line denotes hourly dry bulb temperatures from the future-shifted file produced using projected changes between the past (1991–2020) and the 2060s (2051–2080) under the moderate SSP2 4.5 emissions scenario. The lower panel displays the temperature morphing factors for this same 2060s period. The black line denotes the additive changes in daily average temperature *∆[D_d_(i)]*. The yellow line represents the changes in the diurnal temperature range that result from the relative morphing factor *α[T_d_(i)]*.

Morphing factors for these four variables are obtained from an ensemble of CMIP6 climate models, with projections obtained from the CMIP6 GCMs simulations directly and from statistically downscaled temperature simulations obtained using those same GCMs (CanDCS-U6). Application of the morphing factors to produce future hourly series is first done separately for each GCM, yielding an ensemble of 18 future-shifted series. These series are averaged to produce a single hourly series for each variable that is included in the future-shifted CWEC2020 file. Fig. 2 displays an illustration of the morphing method applied to create future-shifted hourly dry bulb temperatures for the CWEC2020 file at Vancouver International Airport. At this location, the ensemble average projections for the 2060s under a moderate emissions scenario (SSP2 4.5) yield increases in daily average temperatures of between 2.3 °C to 2.9 °C and increases in the diurnal temperature range of between 0.25 °C to 0.5 °C during July and August. Applying these changes to the present-day CWEC2020 dry bulb temperature series (Fig. 2 top panel, black line), yields the future-shifted hourly dry bulb temperature series (Fig. 2 top panel, red line) representing the typical conditions expected during the 2060s (2051–2080) period.

## Limitations

Within the future-shifted files, only four of the variables (dry bulb temperature, dew point temperature, relative humidity, and surface pressure) are adjusted. Hourly series for other variables are not changed either due to projections being unavailable from CMIP6 GCMs or from low confidence in the magnitude and direction of future changes for certain variables. The future-shifted files are provided for five fixed future 30-year intervals spanning the 2040s to 2080s, which may not align exactly with design timelines for different building applications. In the morphing method used to produce the files, while magnitudes of values in the time series are adjusted, the hour-to-hour sequencing of events is preserved from the existing file. Thus, the frequency and duration of persistent events such as heat waves or cold spells remain largely unchanged in the future-shifted files.

## Ethics Statement

The authors have read and followed the ethical requirements for publication in Data in Brief and confirmed that the current work does not involve human subjects, animal experiments, or any data collected from social media platforms.

## CRediT authorship contribution statement

**Stephen R. Sobie:** Conceptualization, Methodology, Investigation, Software, Data curation, Visualization, Writing – original draft. **Charles L. Curry:** Conceptualization, Methodology, Investigation, Writing – review & editing.

## Data Availability

ZenodoFuture-Shifted Weather Files for Canada using Climate Projections from CMIP6 (Original data).
